# Tomato Endophytic Bacteria Composition and Mechanism of Suppressiveness of Wilt Disease (*Fusarium oxysporum*)

**DOI:** 10.3389/fmicb.2021.731764

**Published:** 2021-10-15

**Authors:** Zeyu Zhang, Ji Li, Zengqiang Zhang, Youzhou Liu, Yuquan Wei

**Affiliations:** ^1^College of Resources and Environmental Science, China Agricultural University, Beijing, China; ^2^College of Natural Resources and Environment, Northwest A&F University, Xianyang, China; ^3^Institute of Plant Protection, Jiangsu Academy of Agricultural Sciences (JAAS), Nanjing, China

**Keywords:** endophytic bacteria, 16S rRNA gene, *Bacillus* antagonism, *Fusarium oxysporum*, suppressiveness

## Abstract

Tomato wilt disease, caused by the *Fusarium oxysporum* is an ever-increasing threat for agricultural production, and unreasonable fertilization and pesticide abuse caused environmental challenge. Increasing evidence suggested that microbiomes or those associated with crops, played key roles on plant health. Plant disease dynamics were affected by multiple biotic and abiotic factors including phytopathogen population density, the genetic type of the pathogen and the host, in particular, the composition and assembly of the host-associated microbiome. However, it was unclear how pathogen invasion interaction and correlate with endophytic bacterial communities in natural field conditions. To study this, we sampled temporally the tomato plants that were exposed to *F. oxysporum* invasions over one crop season. High-throughput sequencing were performed to explore the correlation between agricultural practice, pathogen invasion, and endophytic microbiota communities. Results showed that pathogen invasion had clear effect on the endophytic and a strong link between increased pathogen densities and reduced abundance of *Bacillus* sp., which are crucial taxonomy for suppressiveness to *F. oxysporum in vitro* and in greenhouse condition. In summary, monitoring the dynamics of endophytic bacteria communities and densities of pathogen could thus open new avenue for more accurate disease diagnostics and high-efficiency screening antagonisms methods in the future, and our results will broaden the agricultural view of beneficial microbiota as biological control agents against plant pathogen.

## Introduction

Tomato wilt disease, caused by the *Fusarium oxysporum*, poses a growing threat to agricultural production, and the unreasonable fertilization and pesticide abuse used to control this disease has caused an environmental challenge. With the demand for policy regarding the reduction of fertilizer and pesticides, there is an urgent need to reduce the use of environmentally unfriendly pesticides and agrochemicals (Bautista-Baños et al., 2013; Wei et al., 2019). Application of beneficial microbial inoculants can increase crop yields and resist the invasion of pathogens. In addition, increasing cases illustrate that soil microbiomes or those associated with plants play key roles in plant health (Shen, 1997; Curtis et al., 2004; Chen et al., 2018). Endophytic bacteria that can inhabit plant tissues without causing disease has been suggested as a viable alternative to reduce crop disease level (Mazurier et al., 2009). Increasing evidence suggests that taxonomic groups such as Bacteroidetes, Acidobacteria, Firmicutes, and Proteobacteria are involved in biocontrol of soil-borne diseases (Zhang et al., 2009; Mendes et al., 2011; Trivedi et al., 2012; Garciasalamanca et al., 2013; Donn et al., 2015). Various suppressive mechanisms were reported, mainly including the improvement of microbe-mediated pathogen suppression and inducing the plant immune practice (Hu et al., 2016). Specifically, antibiosis, competition, and elicitation of induced systemic resistance (ISR) contributes indirectly to disease suppression (Niu et al., 2017; Kwak et al., 2018). Especially, many antagonistic taxonomies including *Bacillus* spp., *Streptomyces* spp., *Trichoderma* spp., and *Pseudomonads aeruginosa* inhibited soil borne diseases by producing the antibiotics: 2,4-diacetylphloroglucinol, surfactin, fengycin, and antitoxin, etc. (Arrebola et al., 2010; Sandhya et al., 2010; Wang et al., 2019). Fan et al. (2017) found that inoculation of *Bacillus subtilis* 9407 can suppress the bacterial fruit blotch, for instance, surfactin antibiotic mediated the pathogen suppression.

With the development of next-generation sequencing technology, the composition and assembly of plant microbiome dynamics on plant health have been studied for several decades, which might be generally influenced by the genetic type of the host, and temporal and meteorological variation (Dodds and Rathjen, 2010). However, little is still known about how endophytic bacterial communities, pathogen density dynamics, and agricultural manipulation affect how plant disease takes place, that is, how these biotic and abiotic factors interact and correlate with each other in regulating plant disease dynamics in greenhouse conditions. Given the complex interaction between the endophytic bacterial, soil physical and chemical properties, and pathogen dynamics above mentioned, we set out to study the following two key hypotheses:

(i) To what extent do plant pathogen invasion and agricultural practice influence endophytic bacterial communities?
(ii) What are the underlying suppressive taxonomics in the endophytic bacterial communities?

Here, we used comprehensive methods, including greenhouse experiments, high throughput sequencing, and artificially created co-culturing of select (two or more) species assay to study the correlation between agricultural practice, pathogen invasion, and endophytic microbiota communities, and constructing suppressive endophytic bacterial communities. The endophytic bacterial communities under four different crop phases (seedling phase, flowering phase, fruiting phase, and harvesting phase) and different healthy conditions (initial health, final health, and diseased), agricultural practices (conventional practice, organic practice) were explored by using 16S amplicon sequencing in this study. Results showed that pathogen invasion had a clear effect on the endophytic bacterial communities, and *Bacillus* sp. were enriched in the healthy tomato samples at the seedling phase, which implied a preventive-protective effect of *Bacillus*. We also found an antagonism effect on the *F. oxysporum* both in the vitro and vivo conditions. Our results will help to broaden the agricultural applications of synthetic microbial communities as biological control agents against plant pathogens.

## Materials and Methods

### Experimental Field Site, Sampling Regime, and Soil Physicochemical Properties

The field experiment was located in Quzhou county, Hebei province (36°52′N, 115°01′E). The long-term trail treatment included organic, integrated, and conventional farming practices. Details on the agricultural management in each greenhouse were as described by Han et al. (2019), briefly, organic farming practices were characterized by the application of compost, as well as biocontrol for the plant disease, while conventional farming followed the local traditional farming style for greenhouse vegetable production and used chemical fertilizers. Both organic (ORG) and conventional (CON) greenhouse practices were selected with relatively high disease incidence of Tomato *Fusarium oxysporum* wilt (30% disease incidence for organic greenhouse during crop season, and 50% disease incidence for conventional greenhouse during crop season). Ten healthy tomato samples were randomly collected at the organic and conventional greenhouses at the seedling phase. Tomato showed symptoms of wilting and approximately 50% of plants showed clear symptoms of *Fusarium* wilt by the end of the crop season (from 10 weeks from the transplantation), thus, 10 healthy and 10 diseased plants were randomly collected from the organic and conventional greenhouse, and sampling finished after 12 weeks. Each sample was divided into two parts, aboveground (stem) and belowground (root), after the transplantation, thus 30 tomato plants (60 samples) were collected in total (Supplementary Figure 1). Physicochemical properties of soil such as the content total nitrogen, organic matter, ammonia, and pH were determined according to standard protocols (Wang et al., 2020), and soil physicochemical properties were ordinated by Excel (v.2019) and SPSS (v.19).

### qPCR Quantification of *Fusarium oxysporum* Densities

*Fusarium oxysporum* densities were determined with qPCR by using primers targeting the *foC* gene (forward primer: 5′-AACMGGATTAGATACCCKG-3′ and reverse primer: 5′-GCACTGGCATATA-3′, KamaID, Sharma et al., 2004). The qPCR was carried out with Applied Biopractices 7500 Real-Time PCR Practice (Applied Biopractices, CA, United States) by using the SYBR Green I fluorescent dye detection in 20-μl volumes containing 10 μl of SYBR Premix Ex Taq (TaKaRa Biotech. Co., Japan), 2 μl of template, and 0.5 μl of both forward and reverse primers (10 mM each). The qPCR was performed by initially denaturing for 30 s at 95°C with subsequent cycling 40 times with a 5 s denaturizing step at 95°C. The protocol was followed by a 34 s elongation/extension step at 60°C and with a melt curve analysis for 15 s at 95°C followed by 1 min at 60°C and finally for 15 s at 95°C. Melting curves were obtained based on a standard protocol and used to identify the characteristic peak of PCR product (160 bp). Three independent technical replicates were used for each sample.

### DNA Extraction and 16S rRNA Gene Amplicon High Throughput Sequencing

All samples’ surface sterilization for removing the epiphyte was performed as previously described (McPherson et al., 2018), until no bacterial growth was detected after inoculating the roots on R_2_A agar plate at 30°C for 7 days, as well as no amplification of 16S rRNA gene observed in the final wash water as template DNA.

Total microbial community DNA from samples was extracted using a Fast DNA spin Kit for soil (MP, Biomedicals, Santa Ana, Carlsbad, CA, United States). 16S rRNA gene fragments was amplified with the universal primers 799F (5′-GTGCCAGCMGC CGCGGTAA-3′) and 1193R (5′-CCCCGYCAATTCMTTTRA GT-3′) fused with a unique barcode (Liu et al., 2021). Gel-purified PCR products were mixed with equal molar following illumina sequencing using the platform of Hiseq2500.

### Bioinformatic Analysis

The concentrations of the purified amplicon products were determined using QuantiFluor-ST (Promega, WI, United States) and sequenced on an Illumina MiSeq platform at Guangzhou Meige Biological Technology Co., Ltd.

All sequence reads were assigned to each sample based on designed primer and barcodes and the technical regions were trimmed for the following analyses. Sequence data were processed with the UPARSE pipeline (Edwards et al., 2015). High quality sequences (Chimeras were removed using UCHIME, Liu et al., 2021) were used for downstream analyses. Generation of the representative sequences and taxonomic OTU feature table obtained were then analyzed using Mothur (Schloss et al., 2009). Classification and identification of representative OTUs were processed with RDP naïve bayes classifier version 16 (Liu et al., 2019). Shannon and Ace indices were calculated in an algorithm similar to analysis to mitigate the biases caused by low quality sequences (Zhang et al., 2018; Ding et al., 2019; Wang et al., 2020). Community composition of endophytic bacterial visualized by PCoA analysis based on pairwise Bray-Curtis distance was calculated from the level of relative abundance of OTUs, and the effects of plant growth phase, treatment, and plant health condition on bacterial communities based on weighted_unifrac distance by PERMANOVA test, and all statistical analyses were performed with the R 3.1.21 software (R Core Team, 2013). Redundancy analysis (RDA) was used to analyze the correlation between the endophytic bacterial community, soil physicochemical properties, and environmental factors, and the correlation network was performed to compare the bacterial interactions at the OTU level in different treatments. Network analysis and visualization was performed based on Cytoscape software (version 3.4.0, Segata et al., 2011).

Linear discriminant analysis and a significance test were used to explore the most discriminating OTUs between health and diseased samples (Bastian et al., 2009). Three screening criteria were used:

(i) Linear discriminant analysis with a score of ≥4 (health condition relative to diseased condition).
(ii) Fold change ≥ 3.5 (health condition relative to diseased condition).
(iii) Significance test with *P* < 0.05.

The raw sequencing reads were deposited in the NCBI Sequence Read 186 Archive (SRA) under the accession number PRJNA764343 (SAMN 187 21500483).

### Isolation, Screening of Endophytic Antagonists for Tomato Wilt Disease (*Fusarium oxysporum*)

To isolate the endospheric microbes, samples were washed thrice with sterilized PBS buffer (Na_2_HPO_4_ 1.42 g/L; KH_2_PO_4_ 0.24 g/L; NaCl 8 g/L; KCl 0.2 g/L; 0.01%, pH 7.4) shaken (170 rpm) for 2 h at 30°C. After centrifugation at 1,200 rpm at 4°C, all samples were washed until soil particles were completely removed. Then the washed roots and stems were cut up in pieces and a subsample of root tissue, representative of whole plant system, was collected and placed on the R_2_A medium. All bacteria were isolated by picking colonies, and each isolate was randomly selected by Sanger sequencing analysis, and sequence reads were aligned using BLASTn^1^, and the closest match was identified. Antagonism bacterial screening for *F. oxysporum* was based on co-culture in the plate as described (Liu et al., 2011).

### Culture-Dependent Assessment of Antimicrobial Activity of *Bacillus* and *Pseudomonas* Bacteria Against *Fusarium oxysporum* Under Greenhouse Condition

Five antagonists (*Bacilllus megaterium, Bacillus subtilis, Bacillus amyloliquefaciens, Pseudomonas aeruginosa* and *Pseudomonas putida*, Supplementary Table 1) were selected from healthy tomato samples in this study. Co-cultures were performed to demonstrate antagonistic effects of each of the five selected isolates against *F. oxysporum* according to Paidhungat et al. (2000). When tomato seedlings were 20 days old, ten milliliters of the suspension antagonists (approximately 10^8^ spores/mL) were inoculated, then challenged with *F. oxysporum* spores (approximately 10^7^ spores/mL) and the disease incidence was evaluated 7 days after inoculation according to the Li et al. (2019). Three pots were taken and all six seedlings in each pot were used as a replicate for each treatment.

## Results

### Changes in the Soil Physiochemical Properties During the Field Experiment

**FIGURE 1:**
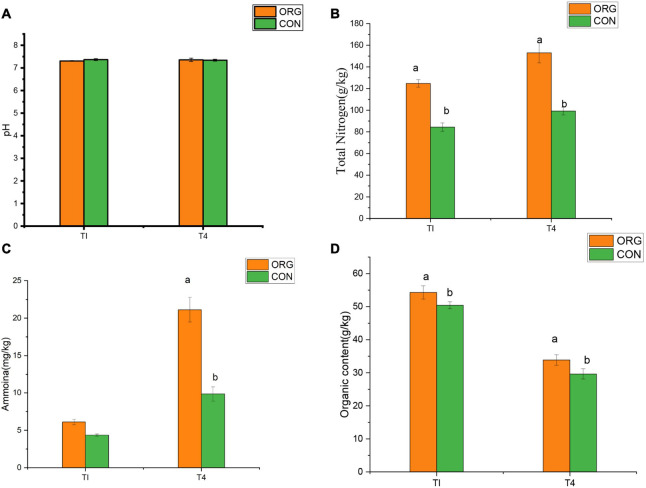
Discrimination of physiochemical soil properties during the field experiment. **(A–D)** Differences in major physiochemical soil properties between seedling phase and fruiting phase (averaged over time). **(A)** pH, **(B)** total nitrogen, **(C)** ammonia content, and **(D)** organic content. Different letters denote dramatically significant (*p* < 0.01). TI, seedling phase; T4, fruiting phase.

To explore the variation of soil physiochemical properties in the greenhouse, four physicochemical properties were measured during the seedling phase and fruiting phase. In addition, soil physiochemical properties were different in two soil types. The results showed that concentration of total nitrogen and organic content of the organic greenhouse were significantly higher than those in the conventional greenhouse (*p* < 0.05, ANOVA, Tukey HSD; Figures 1A–D).

### Disease Development and *Fusarium oxysporum* Density Dynamics During the Field Experiment

**FIGURE 2:**
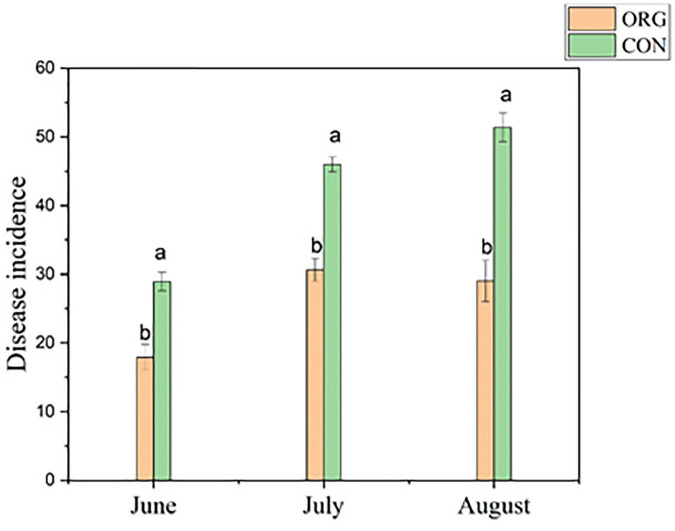
Tomato *Fusarium* wilt disease incidence from June to August in 2019 during the field experiment (*N* = 15).

Pathogens could be detected in all tomato rhizosphere soil samples since the beginning of the sampling and pathogen abundance varied from 10^1.45^ to 10^9.23^ copy of *foC* gene per gram of soil. As tomato *Fusarium* wilt disease showed visible signs at fruiting and harvesting phase, thus disease incidence was observed in June, July, and August. Results showed that disease incidence in the organic greenhouse was about 31.2%, while that of the conventional greenhouse was about 49.8% (Figure 2A), and disease incidence was less severe in the organic practice greenhouse compared to that in the conventional practice greenhouse (*p* < 0.05, ANOVA, Tukey HSD; Figure 2). Intriguingly, a relatively high density of pathogens was observed in either the organic or conventional diseased tomato rhizosphere root soil, specifically, the density of *F. oxysporum* (*foC* gene copy number/g rhizosphere soil) reached about 10^8^ copy number/g in the diseased tomato rhizosphere root soil, while 10^2^ copy number/g rhizosphere root soil in the healthy tomato samples (Table 1).

**TABLE 1:** Tomato rhizosphere *Fusarium oxysporum* pathogen density dynamics from seedling phase (March) to harvesting phase (August) in 2019 during the field experiment (*N* = 10).

**Group**	***Log_10_foC* copy number (g/rhziosphere soil)**
TIOH	2.103 ± 0.42 d
TICH	2.77 ± 0.49 cd
T3CH	2.924 ± 0.61 c
T3CI	8.018 ± 0.80 a
T4OH	2.357 ± 0.03 d
T4OI	6.005 ± 0.49 b

*TIOH stands for initially healthy plants in the organic system greenhouse, T4OH stand for healthy plants in the organic system greenhouse. T4OI stand for diseased plants in the organic system greenhouse. TICH stand for initially healthy, T3CH stand for healthy at last, and T3CI stands for diseased at last in the conventional greenhouse.*

*T1, seedling phase; T3, fruiting phase; T4, harvesting phase.*

*Different letters denote dramatically significant (*p* < 0.01).*

### Healthy Tomato Endosphere Microbiota Have a Distinct Structure

The density dynamics of the most abundant endophytic bacterial phyla differed significantly between the healthy and diseased samples, and results showed that the majority of phyla belonged to Proteobacteria accounting for 41.58%, followed by Firmicutes (26.15%), while Bacteroidetes and Actinobacteria accounted for 20.2 and 11.82%, respectively, in the initial healthy tomato samples, and the most striking results showed that Firmicutes decreased intensely to 3.55% at diseased samples under the organic practice greenhouse. The similar results were also observed at the conventional manipulation greenhouse. Proteobacteria (42.20%) and Firmicutes (29.15%) are the main phyla in the initial healthy tomato samples, and Firmicutes decreased dramatically to 6.43% in the diseased samples in the conventional practice greenhouse (Supplementary Figures 2, 3).

**FIGURE 3:**
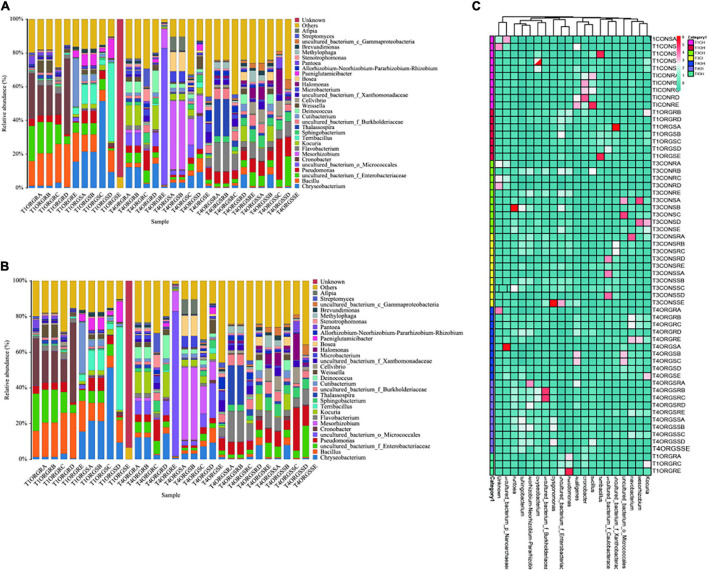
Changes in the endophytic microbiome composition and diversity during the field experiment. **(A)** The relative bacterial density dynamics in initial, healthy, and diseased tomato endophytic samples in the organic system greenhouse. **(B)** The relative bacterial density dynamics in initial, healthy, and diseased tomato endophytic samples in the conventional system greenhouse. **(C)** Heatmap analysis of endophytic microbiome composition during the field experiment. T1OH stands for initially healthy, T4OH stands for healthy at last, T4OI stands for diseased at last. TICH stands for initially healthy, T3CH stands for healthy at last, T3CI stands for diseased at last in the conventional greenhouse, TIOH stands for initially healthy plants in the organic system greenhouse, T4OH stands for healthy plants in the organic system greenhouse, and T4OI stands for diseased plants in the organic system greenhouse.

**FIGURE 4:**
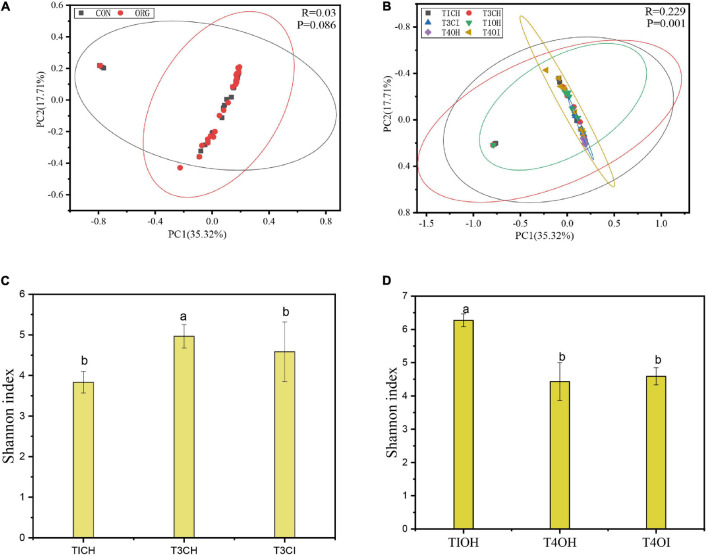
**(A)** PCoA of bacterial microbiota using Bray–Curtis distance for organic and conventional agricultural practice. ORG stands for organic greenhouse. CON stands for conventional greenhouse. **(B)** PCoA of bacterial microbiota using Bray–Curtis distance for healthy and diseased plants. **(C)** OTU bacterial Shannon diversity index for different treatments at organic greenhouse. **(D)** OTU bacterial Shannon diversity index for different treatments at conventional greenhouse. TIOH stands for initially healthy plants in the organic system greenhouse, T4OH stands for healthy plants in the organic system greenhouse. T4OI stands for diseased plants in the organic system greenhouse. TICH stands for initially healthy, T3CH stands for healthy at last, and T3CI stands for diseased at last in the conventional greenhouse. T1, seedling phase; T3, fruiting phase; T4, harvesting phase. Different letters denote dramatically significant (*p* < 0.01).

Taxonomic dynamics of the most abundant endophytic bacterial genera differed significantly between the healthy and diseased samples. In general, the relative abundance of the *Bacillus*, *Cronbacter*, and *Weeksellaceae* clearly decreased in the diseased compared to healthy plant samples, while *Sphingobacterium*, *Flavobacterium*, *Burkholderiaceae*, and *Xanthobacteraceae* increased in the diseased plant samples in the organic greenhouse. Similar trends occurred in the conventional greenhouse, crucially, *Bacillus* sp. were observed in the relative high abundance of bacterial genus at healthy samples in the seedling phase, and the density of which decreased in the diseased tomato samples (Figures 3A,B). Interestingly, low abundance of *Bacillus* was observed in the healthy samples at the late crop season (Figure 3C). Diversity decreased in diseased samples compared with healthy samples regardless of organic or conventional greenhouse (*p* < 0.05, ANOVA, Tukey HSD), Specifically, the increase in pathogen densities was associated with reduced endophytic bacterial diversity both in the organic and conventional greenhouse (Shannon index, Figures 4C,D).

### Pathogen Invasion Disrupts the Endophytic Bacterial Composition

**FIGURE 5:**
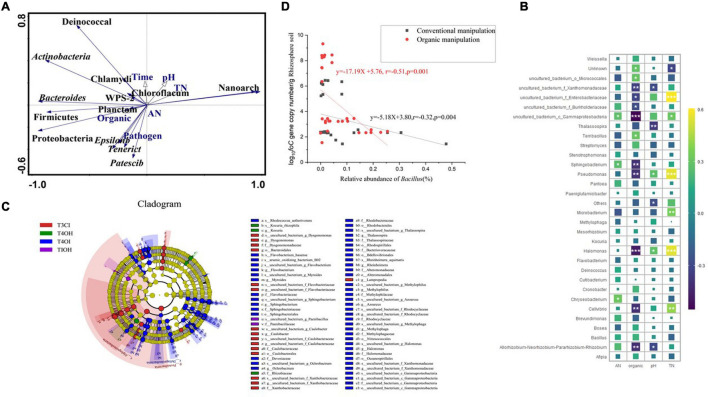
**(A)** The relationship among the bacterial community, environmental parameters. Redundancy analyses (RDA) of the correlation between the bacterial community (Phylum level) and soil physicochemical properties. Gray lines represent significant factors and Blue lines represent key Phyla. **(B)** Correlation (Pearson’s coefficient) heat map between key rhizosphere soil bacterial community (genus level) and soil physicochemical properties of rhizosphere soil. *Denotes significance (*P* < 0.05, FDR adjust), and ** denotes significance (*P* < 0.01, FDR adjust). **(C)** Lefse analysis discrepancy in Endophytic bacterial taxonomy of Tomato between Healthy and Diseased Plants in the organic system greenhouse (LDA > 3.5, FD > 2, *P* < 0.05). **(D)** Correlation between log_10_*foC*gene copy number/g rhizosphere soil and relative abundance of *Bacillus* sp. in the organic greenhouse and conventional greenhouse.

PCoA analysis showed that pathogen invasion had strong effects on endophytic bacteria composition, healthy and diseased samples clustered with two axes regardless of organic or conventional agricultural practice (*R*^2^ = 0.229, *P* = 0.001, PERMANOVA analysis; Figures 4A,B). To investigate the diversity of changes occurring in microbial community structures at the phylum level, redundancy analysis (RDA) was conducted. Results showed that all of the edaphic variables explained up to 56.28% of the variance, with the first axis explaining 36.73% of variation and the second axis explaining another 19.55% (Figure 5A). RDA plots showed that pathogens might be key physicochemical factors in assembling the microbial community structure of the tomato endophytic microbiome, and Firmicutes showed positive reaction to pathogen invasion. In addition, the correlation heatmap showed that the content of total nitrogen and organic had a positive influence on Proteabacteria and Gemmatimonadetes (Figure 5B).

### Microbial Biomarkers in Different Treatments and Healthy Conditions

Lefse analysis discrepancy based on datasets of healthy and diseased plants revealed that *Bacillus* sp. were enriched in the healthy tomato samples during the seedling phase (LDA > 3.5, FD > 2, *p* < 0.05), while *Sphingobacterium*, *Flavobacterium*, *Burkholderiaceae*, and *Xanthobacteraceae* were enriched in the diseased tomato samples. Similar results occurred in the conventional greenhouse, and Lefse analysis also showed that discrepancy decreased with plant growth between each treatment (Figure 5C). Negative correlation between pathogen density dynamics and relative abundance of *Bacillus* sp. were observed both in the organic and conventional greenhouse based on linear regression (Figure 5D).

### Seedling Phase Healthy Tomato Steered a Highly Connected Association Network

**FIGURE 6:**
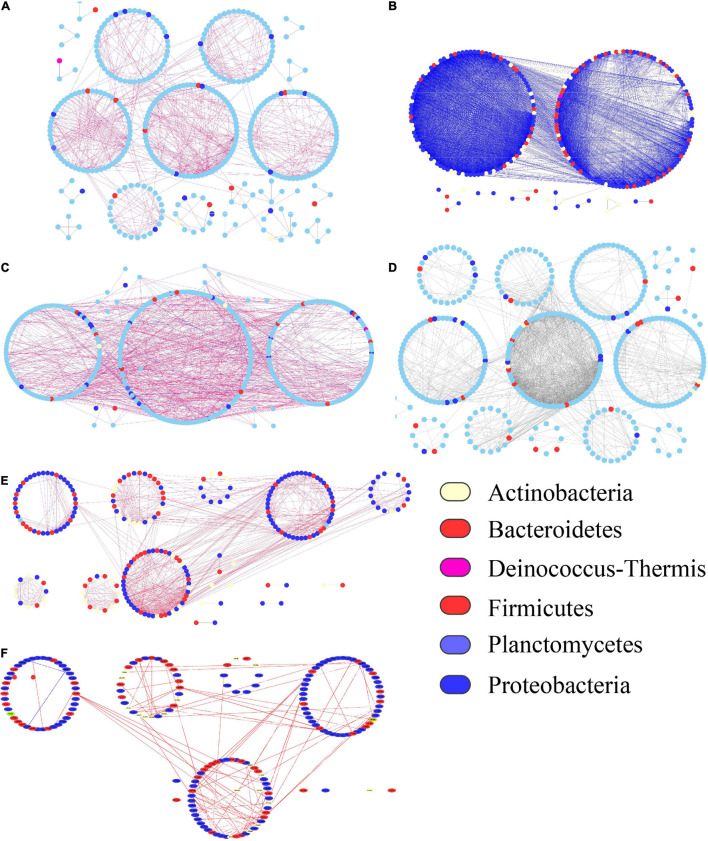
Visualized networks of microbial co-occurrence patterns. **(A)** T1OH, **(B)** T4OH, **(C)** T4OI, **(D)** TICH, **(E)** T3CH, and **(F)** T3CI. TIOH stands for initially healthy plants in the organic system greenhouse, T4OH stands for healthy plants in the organic system greenhouse, T4OI stands for diseased plants in the organic system greenhouse, TICH stands for initially healthy, T3CH stands for healthy at last, and T3CI stands for diseased at last in the conventional greenhouse. T1, seedling phase; T3, fruiting phase; T4, harvesting phase.

Microbial networks were generated to depict the co-occurrence patterns of the microbial community in different treatments and plant healthy statuses. The results showed that pathogen invasion disrupted significantly simplified microbial associations as compared with the healthy tomato which had a complexity of microbial associations (Figure 6). Specifically, the average degrees in networks markedly reduced from 39.272 in the seeding phase organic endophyte group (T1OH) to 4.186 and 2.664 in the fruiting phase healthy organic endophyte group (T4OH) and diseased organic endophyte group (T4OI) respectively. Similarly, the average degrees in networks markedly reduced from 7.708 in the seeding phase organic endophyte group (T1CH) to 7.269 and 2.93 in fruiting phase healthy organic endophyte group (T3CH) and diseased organic endophyte group (T3CI) respectively (Table 2). In comparison to the diseased and healthy plants, we further found that the dominant keystone taxa in the diseased and healthy treatments were mainly represented by potential plant beneficial or antagonistic bacterial taxa like Actinobacteria, Proteobacteria, and Firmicutes (Table 2).

**TABLE 2:** Topological indices of microbial co-occurrence network in different treatments.

**Network indexes**	**TIOH**	**T4OH**	**T4OI**	**TICH**	**T3CH**	**T3CI**
Total nodes	431	527	293	445	238	172
Total links	4,693	702	902	1,715	865	252
*R* square of power-law	0.117	0.896	0.826	0.857	0.767	0.917
Average degree (avgK)	39.272	4.186	2.664	7.708	7.269	2.93
Average clustering coefficient (avgCC)	0.692	0.189	0.163	0.383	0.424	0.135
Average path distance (GD)	3.839	11.174	4.891	5.256	4.639	5.413
Geodesic efficiency (E)	0.421	0.119	0.24	0.239	0.287	0.24
Harmonic geodesic distance (HD)	2.378	8.373	4.16	4.176	3.488	4.172
Maximal degree	86	13	28	53	35	18
Nodes with max degree	OTU221; OTU70	OTU82227	OTU14044 OTU6001	OTU339	OTU808	OTU63877
Centralization of degree (CD)	0.198	0.02	0.056	0.102	0.118	0.089
Maximal betweenness	3,288.704	20,108.758	9,076.833	29,377.169	5,391.234	2,288.825
Nodes with max betweenness	OTU68	3,694,599	OTU771	OTU2725	OTU98	OTU34635
Centralization of betweenness (CB)	0.107	0.136	0.093	0.292	0.183	0.143
Maximal stress centrality	37,949,354	58,862	79,052	279,209	179,439	12,783
Nodes with max stress centrality	OTU68	334,296	OTU771	OTU2725	OTU98	OTU63877
Centralization of stress centrality (CS)	0.107	0.136	0.093	0.292	0.183	0.143
Maximal eigenvector centrality	37,949,354	58,862	79,052	279,209	179,439	12,783
Nodes with max eigenvector centrality	OTU68	334,296	OTU771	OTU2725	OTU98	OTU63877
Centralization of eigenvector centrality (CE)	1,240.599	0.396	0.802	2.763	6.208	0.807
Density (D)	0.129	0.342	0.348	0.203	0.269	0.366
Reciprocity	1	1	1	1	1	1
Transitivity (Trans)	0.776	0.388	0.132	0.534	0.492	0.287
Connectedness (Con)	0.811	0.994	0.988	0.98	0.961	0.98
Efficiency	0.811	0.994	0.988	0.98	0.961	0.98
Hierarchy	0	0	0	0	0	0
Lubness	1	1	1	1	1	1

*TIOH stands for initially healthy plants in the organic system greenhouse, T4OH stands for healthy plants in the organic system greenhouse, T4OI stands for diseased plants in the organic system greenhouse, TICH stands for initially healthy, T3CH stands for healthy at last, and T3CI stands for diseased at last in the conventional greenhouse.*

*T1, seedling phase; T3, fruiting phase; T4, harvesting phase.*

### *Bacillus* sp. Exhibited High Biocontrol Efficacy on *Fusarium oxysporum*

**FIGURE 7:**
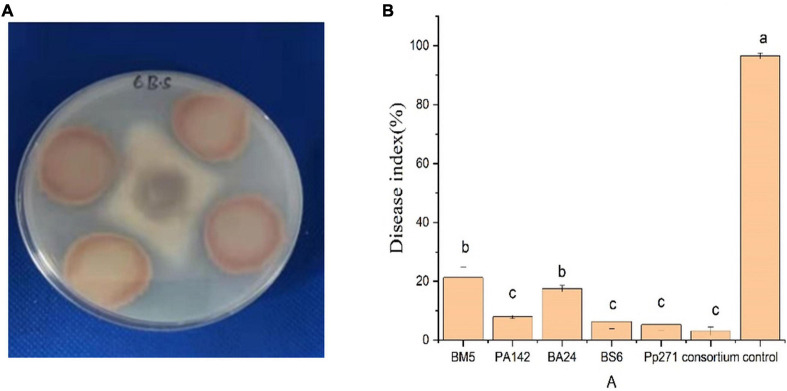
**(A)** Antagonism effect of endophytic bacterial *in vitro*. Strain inoculated on PDA plates, incubated at 30°C for 2 days and then sprayed with the isolated bacteria. **(B)** Bioassay against *F. oxysporum* in the greenhouse condition, *n* = 6, significant difference is denoted by different letters (*P* < 0.05).

Among the antagonists, 14 *Bacillus* strains, 11 Pseudomonas including *Bacillus Megaterium, Bacillus subtilis*, *Bacillus amyloliquefaciens Pseudomonas aeruginosa*, and *Pseudomonas putida* belonging to species (Supplementary Table 1) that are usually reported as biocontrol agents in the specialized literature were observed to have a direct inhibition effect on *F. oxysporum*. Specifically, the inhibition zone of *Bacillus subtilis* 6 reached 2.0 ± 0.26 cm (*p* < 0.05, Figure 7A). *Bacillus Megaterium* 5, *Bacillus subtilis* 6, *Bacillus amyloliquefaciens* 24, *Pseudomonas aeruginosa* 142, and *Pseudomonas putida* 271 were selected to evaluate their antagonistic capacity against pathogens in the greenhouse condition for their desirable inhibition effect on *F. oxysporum in vitro*, and results of the greenhouse bioassay showed that biocontrol effect reached 92.8% (*P* < 0.05, Figure 7B and Supplementary Figure 4).

## Discussion

Here we explored how agricultural practice (organic and conventional agricultural manipulation system), crop season (plant development time), and endophytic bacterial community composition interact with the invasion of *F. oxysporum* in tomato under field conditions. Host-associated microbiome communities have repeatedly been observed, especially between healthy and diseased plant individuals of which discriminative taxonomies are hotspots in the related research (Adesina et al., 2007; van der Heijden and Schlaeppi, 2015; Perez-Jaramillo et al., 2019).

Wei et al. (2018) found no difference in the rhizosphere soil physicochemical properties between healthy and diseased plants, suggesting that abiotic factor may not drive the outbreak of disease. The soil’s physiochemical properties showed clear discrepancy patterns with the agricultural practice in this study, however, only bulk soil physical properties instead of rhizosphere soil of each sampled tomato were determined in this study, and the correlation between rhizosphere soil properties and each sample’s *F. oxysporum* dynamics still needs to be explored.

Clearly, decline was found in the relative abundances of Firmcutes, Bacterioidetes, and Actinomycetes (Supplementary Figures 1, 2), which is in line with other similar results, that is, Firmcutes decline leads to disruption of host-associated microbiome exacerbated *F. oxysporum* invasion (Walters et al., 2018). The final health status of tomato is significantly related to its initial (seedling phase) endophytic bacterial community composition based on Lefse analysis. Temporal pathogen dynamics and endophyte associated with healthy and diseased plants were analyzed with linear regression, and a negative correlation was observed between pathogen density and abundance of *Bacillus* sp., demonstrating that relative abundance of *Bacillus*, especially in the initial growing phase, makes a contribution to the suppression of pathogens (Figure 5B). Among the antagonistic bacteria isolated at the seedling phase in this study, the abundance of *Bacillus* was enriched, reaching 71%, while reaching 29% in the late crop season (Fruiting phase) (Supplementary Figure 5). We conclude that the host endophyte *Bacillus* can provide protection from plant disease in the early crop phase.

Our results support the hypothesis that agricultural practice and pathogen invasion had a clear effect on the endophytic bacteria communities in line with other research (Trivedi et al., 2012). Complexity of microbial associations, and the resulting higher percentage of co-occurrence in the networks during pathogen invasion, indicates lower potential competition for resources and predators and an increased cooperation within the network (Figure 6).

Similar results showed that development of dysbiosis, a microbial imbalance associated with the increased density of bacterial pathogen, could thus also be important in explaining the bacterial wilt disease dynamics (Trivedi et al., 2017; Shi et al., 2020). Related research showed that dysbiosis of the phyllosphere microbiota was recently reported to cause disease on Arabidopsis leaves, and reduction of Firmicutes caused by an increase of the Proteobacteria population was the main driver of this dysbiosis (Chen et al., 2020). Dysbiosis of microbiota communities can also trigger loss predicted community functioning module based on metagenomics analysis (Wei et al., 2018). Our results also demonstrated that *Bacillus* sp. and *pseudomonas* sp. have an antagonist effect on the *F. oxysporum* both in the vivo and vitro conditions (Figure 7), which is in line with previous study which found that Firmicutes taxa, *Bacillus*, *Flavobacterium*, and *Streptomyces* establish disease suppression by a single strain or SynCom method *via* antagonistic effect (Kim et al., 2016; Xu et al., 2018; Wang et al., 2020). However, mechanisms of *Bacillus* sp. and *Pseudomonas* sp. in the suppression of *F. oxysporum* still need to be explored.

This study was based on a long-term greenhouse experiment in which several agricultural practices, including crop rotation, tillage, and irrigation, were the same for all three farming systems since 2002. On the basis of our results, we recommend that the current method to control plant diseases in agricultural systems be reconsidered. First, by modifying the composition of the microbial as a whole, instead of focusing on controlling pathogens directly, thus a better solution to control the outbreak of plant diseases can be achieved.

## Conclusion

Pathogen invasion can drive community-wide dynamics in tomato endophytic bacterial composition. *Bacillus* sp. which is enriched in the seedling phase may play key role for the suppression of *F. oxysporum* based on Lefse analysis. Healthy tomato steered a higher connected association network than that in diseased tomato regardless of organic or conventional agricultural treatment. *Bacillus* and *Pseudomonas* are the dominant taxa in the antagonists of *F. oxysporum* in both of the field conditions.

## Data Availability Statement

The datasets presented in this study can be found in online repositories. The names of the repository/repositories and accession number(s) can be found below: NCBI (accession: PRJNA764343).

## Author Contributions

ZyZ and JL: methodology, validation, formal analysis, writing–original draft, and writing–review and editing. ZqZ, YL, and YW: revising and editing. All authors contributed to the article and approved the submitted version.

## Conflict of Interest

The authors declare that the research was conducted in the absence of any commercial or financial relationships that could be construed as a potential conflict of interest.

## Publisher’s Note

All claims expressed in this article are solely those of the authors and do not necessarily represent those of their affiliated organizations, or those of the publisher, the editors and the reviewers. Any product that may be evaluated in this article, or claim that may be made by its manufacturer, is not guaranteed or endorsed by the publisher.

## Footnotes

1
http://blast.ncbi.nlm.nih.gov

## Supplementary Material

Supplementary Figure 1 Schematic figure of the field sampling system and the experimental design. Treatments have organic and conventional two manipulation greenhouses. Initial healthy samples were collected when the experiment was conducted (4 weeks), and diseased samples were collected when the experiment was conducted (10 and 12 weeks after planting).

Supplementary Figure 2 Changes in the endophytic microbiome composition at the phylum level during the field experiment. The relative bacterial density dynamics in initial, healthy, and diseased tomato endophytic samples in the organic system greenhouse.

Supplementary Figure 3 Changes in the endophytic microbiome composition at the phylum level during the field experiment. The relative bacterial density dynamics in initial, healthy, and diseased tomato endophytic samples in the conventional system greenhouse.

Supplementary Figure 4 Evaluation of disease suppression effect by greenhouse bioassay (*N* = 18).

Supplementary Figure 5 Percentage of different antagonistic bacteria in the Seedling and Fruiting phases.
