# Supplementary figures and images for: Tomato Endophytic Bacteria Composition and Mechanism of Suppressiveness of Wilt Disease (Fusarium oxysporum)
Source: Front Microbiol. 2021 Oct 15;12:731764. doi: 10.3389/fmicb.2021.731764 (PMC8555416; doi:10.3389/fmicb.2021.731764)

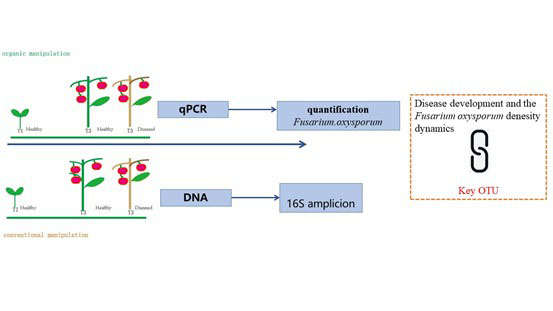

Supplement: Supplementary Figure 1 — Schematic figure of the field sampling system and the experimental design. Treatments have organic and conventional two manipulation greenhouses. Initial healthy samples were collected when the experiment was conducted (4 weeks), and diseased samples were collected when the experiment was conducted (10 and 12 weeks after planting). [file Image_1.JPEG]

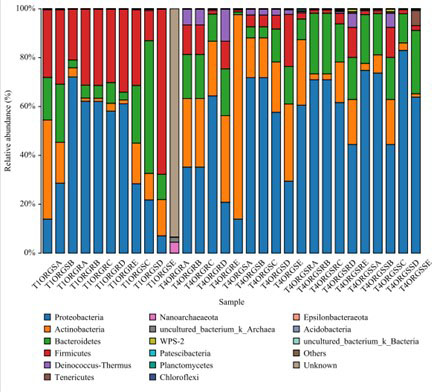

Supplement: Supplementary Figure 2 — Changes in the endophytic microbiome composition at the phylum level during the field experiment. The relative bacterial density dynamics in initial, healthy, and diseased tomato endophytic samples in the organic system greenhouse. [file Image_2.JPEG]

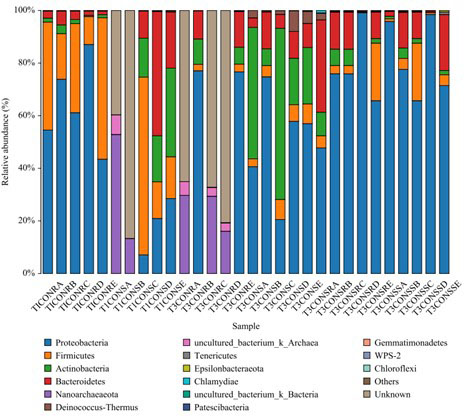

Supplement: Supplementary Figure 3 — Changes in the endophytic microbiome composition at the phylum level during the field experiment. The relative bacterial density dynamics in initial, healthy, and diseased tomato endophytic samples in the conventional system greenhouse. [file Image_3.JPEG]

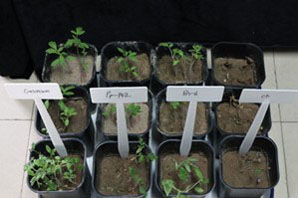

Supplement: Supplementary Figure 4 — Evaluation of disease suppression effect by greenhouse bioassay (N = 18). [file Image_4.JPEG]

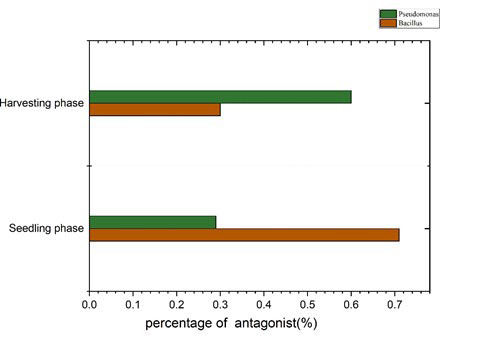

Supplement: Supplementary Figure 5 — Percentage of different antagonistic bacteria in the Seedling and Fruiting phases. [file Image_5.JPEG]
