# Supplementary material for: Tomato Endophytic Bacteria Composition and Mechanism of Suppressiveness of Wilt Disease (Fusarium oxysporum)
Source: Front Microbiol. 2021 Oct 15;12:731764. doi: 10.3389/fmicb.2021.731764 (PMC8555416; doi:10.3389/fmicb.2021.731764)
Supplement: Supplementary file 6 [file Table_1.docx]

Supplemental Table 1 antagonistic taxonomy of endophytic bacterial

| **Strian Number** | **Simliarity strain** | **Representative isolates**  **accession number** | **Similarity** |
| --- | --- | --- | --- |
| **TI_1** | ***Bacillus stratosphericus strain 41KF2a*** | **NR_042336.1** | **100%** |
| **TI_2** | ***Bacillus altitudinis 41KF2b*** | **NZ_ASJC00000000.1** | **100%** |
| **TI_3** | ***Bacillus megaterium* strain ATCC 14581** | [**NR_117473.1**](https://www.ncbi.nlm.nih.gov/nucleotide/NR_117473.1?report=genbank&log$=nuclalign&blast_rank=2&RID=DKHZJC51013) | **100%** |
| **TI_4** | ***Bacillus subtilis* strain DSM 10** | [**NR_027552.1**](https://www.ncbi.nlm.nih.gov/nucleotide/NR_027552.1?report=genbank&log$=nuclalign&blast_rank=1&RID=DKJ4FFE7013) | **100%** |
| **TI_6** | ***Bacillus subtilis subsp. inaquosorum strain BGSC 3A285*** | **MN900587.1** | **100%** |
| **TI_7** | ***Bacillus aerophilus strain 28K*** | **NR_042339.1** | **100%** |
| **TI_8** | ***Bacillus subtilis strain 168*** | **NR_112116.1** | **100%** |
| **TI_9** | ***Pseudomonas mendocina strain ATCC 25411*** | **NR_114477.1** | **100%** |
| **TI_10** | ***Pseudomonas alcaligenes strain IAM 12411*** | **NR_043419.1** | **100%** |
| **TI_14** | ***Bacillus subtilis subsp. spizizenii strain NRRL B-23049*** | **NR_024931.1** | **100%** |
| **T4_1** | ***Bacillus subtilis subsp. inaquosorum strain BGSC 3A28*** | **NR_104873.1** | **100%** |
| **T4_3** | ***Bacillus piscis strain 16MFT21*** | **NR_165685.1** | **100%** |
| **T4_23** | ***Pseudomonas oryzihabitans strain L-1*** | [***NR_113651.1***](https://www.ncbi.nlm.nih.gov/nucleotide/NR_113651.1?report=genbank&log$=nuclalign&blast_rank=1&RID=DKK0XTZG016) | **100%** |
| **T4_28** | ***Pseudomonas putida strain*** | ***NBRC 14164*** | **100%** |
| **T4_31** | ***Pseudomonas oryzihabitans strain L-1*** | **NR_025881** | **100%** |
